# Practical Pharmacological Treatment of Heart Failure: Does Ejection Fraction Matter Anymore?

**DOI:** 10.3390/jcdd10030114

**Published:** 2023-03-09

**Authors:** Jonathan C. H. Chan, Emily Cowley, Michael Chan

**Affiliations:** 1 Faculty of Pharmacy and Pharmaceutical Sciences, University of Alberta, Edmonton, AB T6G 2R3, Canada; jcchan@ualberta.ca; 2 Department of Medicine, Faculty of Medicine and Dentistry, University of Alberta, Edmonton, AB T6G 2R3, Canada; 3 Department of Pharmacy, University of Alberta Hospital, Edmonton, AB T6G 2B7, Canada

**Keywords:** heart failure, ejection fraction, SGLT2i, beta blocker, spironolactone, ivabradine, cardiovascular disease, dilated cardiomyopathy

## Abstract

Heart failure (HF) is a complex clinical syndrome involving structural and/or functional abnormalities of the heart. Heart failure is often classified based on left ventricular ejection fraction, which serves as a predictor of mortality. The majority of the data supporting disease-modifying pharmacological therapies are from patients with reduced ejection fraction (less than 40%). However, with the recent results from the sodium glucose cotransporter-2 inhibitor trials, there is renewed interest in identifying potential beneficial pharmacological therapies. This review focuses on and includes pharmacological HF therapies across the spectrum of ejection fraction, providing an overview of the novel trials. We also examined the effects of the treatments on mortality, hospitalization, functional status, and biomarker levels to further investigate the interplay between ejection fraction and HF.

## 1. Introduction

Heart failure (HF) is a complex heterogeneous clinical syndrome that includes symptoms and signs as a result of structural changes or functional impairment of ventricular filling [1]. Left ventricular ejection fraction (LVEF) is a common classification framework of HF and is used as a clinical measure for the diagnosis. International societal guidelines for heart failure define heart failure with “reduced” ejection fraction (HFrEF) as less than 40%, “mid-range or borderline” (HFmEF) as 41–49% and “preserved” (HFpEF) as greater than 50% [2,3]. In terms of pharmacological management, high or maximally tolerated target doses of medication are derived from large randomized clinical trials in the HFrEF population. The majority of pharmacological therapies have not had the same magnitude of effect as EF increases.

This state-of-the art review focuses on heart failure management across the EF spectrum with a special focus on heart failure pharmacological therapies. It is intended to engage clinicians and researchers to discuss the latest evidence and challenge the dogmas of heart failure and its relationship with EF.

## 2. Materials and Methods

We reviewed 33 randomized controlled trials pertaining to foundational heart failure therapies from 1987 to 2022. We included angiotensin-converting enzyme inhibitors (ACEi), angiotensin receptor blockers (ARB), beta blockers (BB), mineralocorticoid receptor antagonists (MRA), ivabradine, angiotensin receptor—neprilysin inhibitors (ARNI), and sodium glucose cotransporter-2 inhibitors (SGLT2i). Data were extracted independently by two authors (J.C and E.C) through detailed review of the full texts.

## 3. Results

### 3.1. Angiotensin-Converting Enzyme Inhibitors in Heart Failure

Many years ago, heart failure treatment mainly consisted of diuretics and digoxin to alleviate symptoms [4]. Other pharmacotherapies were not well studied. The CONSENSUS [5], SOLVD 1991 [6], and SOLVD 1992 [7] placebo-controlled trials studied the effects of enalapril in patients with HFrEF or patients with asymptomatic reduced LVEF (Table 1). The CONSENSUS trial was the first trial to examine enalapril in patients with HFrEF. Patients were randomized to either enalapril or placebo. These foundational trials demonstrated the benefit of ACEi in reducing death in HFrEF patients. We identified one trial, PEP-CHF, that examined perindopril in an elderly HFpEF population likely caused by hypertension [8].

### 3.2. Angiotensin Receptor Blockers in Heart Failure

ACEi became the standard HFrEF therapy after the CONSENSUS and SOLVD trials. Subsequently ARB add-on therapy trials were commenced through the Val-HeFT trial that did not identify a survival benefit in HFrEF. We identified several trials in the HFrEF and HFpEF populations (Table 2). Trials were either head-to-head trials comparing ARB to ACEi, the addition of ARB to ACEi, or monotherapy ARB. We identified two HFpEF trials: CHARM-Preserved consisted of ischemic HF etiology, while I-Preserved included patients largely with hypertension.

### 3.3. Sacubitril-Valsartan in Heart Failure

Sacubitril-valsartan has been studied throughout the entire HF spectrum, including one trial in the HFpEF population (Table 3). The most common clinical outcomes studied include CV death and HHF. The benefits of sacubitril-valsartan on CV outcomes were derived from the PARADIGM-HF trial including HFrEF patients with NYHA class II to IV in comparison to enalapril [16]. The PARAGON-HF trial did not identify any meaningful differences between sacubitril-valsartan and valsartan in the mildly reduced and preserved EF patient population (*p* = 0.059) [17]. In terms of safety outcomes, the trials were consistent with identifying worse hypotension with sacubitril-valsartan compared to ACEi or ARB. However, there was either a similar or lower rate of worsening renal function and hyperkalemia with sacubitril-valsartan.

### 3.4. Beta Blockers in Heart Failure

Beta blockers are one of the cornerstone therapies for heart failure reduced ejection fraction; however, beta blocker usage in heart failure preserved ejection fraction was previously not well established. The landmark trials for beta blockers in heart failure with reduced ejection fraction are the MERIT-HF, CIBIS-II, COPERNICUS and US Carvediolol trials (Table 4). Beta blockers have been shown to reduce heart failure for hospitalization and CV mortality. In the HFpEF and HFmEF population, the data for beta blockers were derived from other landmark trials where beta blockers were prescribed concomitantly.

### 3.5. Mineralocorticoid Receptor Antagonists in Heart Failure

Trials utilizing MRA in the heart failure population have been used in preserved and reduced ejection fraction (Table 5). MRA provide significant benefit in HFrEF despite the differences in cut-offs between RALES, EPHESUS and EMPHASIS-HF. EPHESUS differed in that it primarily consisted of acute myocardial infarction patients who displayed HF symptoms on presentation. Of the four included trials, only TOPCAT included those with HFpEF [25]. TOPCAT did not demonstrate any significant reduction in the primary composite endpoint. However, its controversial enrollment led to subsequent post hoc analyses excluding geographic regions.

### 3.6. Ivabradine in Heart Failure

Ivabradine was trialed across HFrEF, HFpEF and HFmEF and demonstrated favorable benefits in the HFrEF population (Table 6). Of note, ivabradine inhibits the I_f_ current in the sinoatrial node and was specifically studied in patients with heart rates ≥70 beats per minute with normal sinus rhythm. Heart rate in HF has been identified as a risk factor associated with poor CV outcomes. At present, ivabradine is recommended as an additional therapy in addition to maximally tolerated beta blockers [3,28]. 

### 3.7. Sodium Glucose Cotransporter 2 Inhibitor in Heart Failure

Sodium Glucose Cotransporter 2 Inhibitors (SGLT2i) were originally used as glucose-lowering drugs. However, since the era of cardiovascular outcome trials, SGLT2i has been shown to confer additional cardiovascular benefits.

The DAPA-HF trial was the first SGLT2i heart failure trial that studied the effect of dapagliflozin in patients with heart failure with reduced ejection fraction (HFrEF) [31]. The dapagliflozin cohort had a lower occurrence of worsening heart failure or cardiovascular death. Both hospitalization for heart failure (HHF) and CV mortality were reduced by dapagliflozin regardless of diabetic status.

The EMPEROR-Reduced trial compared empagliflozin to placebo in HFrEF patients [32]. CV death or HHF was reduced by empagliflozin.

EMPEROR-Preserved was the first trial to study the role of SGLT2i heart failure with preserved ejection fraction (HFpEF) (ejection fraction above 40%) [33]. CV death or HHF were reduced by empagliflozin in both patients with or without diabetes.

The SOLOIST-WHF trial evaluated sotagliflozin, which is both a SGLT2 and sodium glucose cotransporter 1 (SGLT1) inhibitor in patients hospitalized for worsening heart failure, which interestingly included patients with heart failure symptoms [34]. Sotagliflozin reduced cardiovascular death and hospitalization. This trial demonstrated that SGLT2i therapy can be started safely and effectively in patients even after an episode of decompensation [35].

The DELIVER trial is the most inclusive SGLT2i heart failure trial. The trial included both hospitalized patients and outpatients with an ejection fraction of 40% or greater or an improved ejection fraction (previously EF < 40%) [36]. Dapagliflozin was shown to reduce the primary composite endpoint of cardiovascular death or worsening heart failure. Even more impressively, a pooled meta-analysis of the DAPA-HF and DELIVER trials found that dapagliflozin reduced the risk of cardiovascular death (HR, 0.86; 95% CI 0.76–0.97; *p* = 0.01), HHF (RR, 0.71; 95% CI 0.65–0.78; *p* < 0.001), and MACE (HR, 0.90; 95% CI 0.81–1.00; *p* = 0.045) across a whole range of left ventricular ejection fractions, from ejection fractions of 25% to 65% [37].

These impressive benefits irrespective of care setting and patient characteristics continue to strengthen the role of SGLT2i as a foundational heart failure therapy while challenging the relevance of ejection fraction in guiding therapy (Table 7).

## 4. Discussion

HF is a multifactorial disease with several drug therapies targeting the sympathetic and renin-angiotensin-aldosterone system. The majority of HF landmark trials have demonstrated a significant reduction in mortality or CV benefit in HFrEF. However, in HFpEF, only two landmark trials studying SGLT2i found a significant benefit with their primary outcome. The other pharmacological therapies were unable to replicate the same benefit in HFpEF. We reviewed the pharmacological HF landmark trials, summarizing the results and attempted to determine if EF truly matters.

### 4.1. Ejection Fraction as a Dichotomous Variable

**Figure 1:**
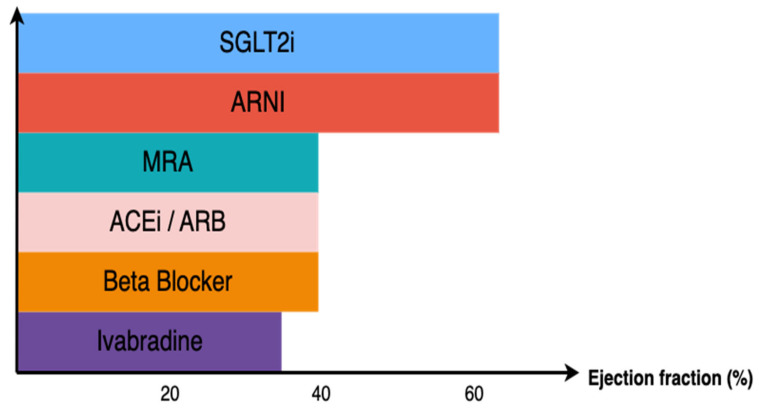
Foundational heart failure therapy consisting of Sodium Glucose Cotransporter 2 Inhibitors (SGLT2i), mineralocorticoid receptor antagonists (MRA), angiotensin-converting enzyme inhibitors (ACEi), angiotensin receptor blockers (ARB), angiotensin receptor-neprilysin inhibitors (ARNI), beta blockers and ivabradine and their respective ejection fraction spectrums.

The classification of HF using cutoffs can help guide clinicians and simplify the implementation of pharmacological therapies, especially in HFrEF (Figure 1). Treatment advocated by international societal guidelines of HFpEF primarily consists of symptom management and optimization of comorbid conditions with weak recommendations for MRA, ARB and ARNI to decrease hospitalizations. Candesartan (CHARM-Preserved), perindopril (PEP-CHF) and irbesartan (I-PRESERVE) were investigated in the HFpEF population, and this resulted in no significant differences in their respective primary composite endpoints [14,15]. TOPCAT was the largest study, with approximately 3500 patients, investigating spironolactone and HFpEF clinical outcomes [25]. It was unable to demonstrate significant benefits regarding death from CV causes, aborted cardiac arrest or HHF. These findings are similar to those from PARAGON-HF, which investigated sacubitril-valsartan [17]. Additionally, in the exploratory subgroup analyses of CHARM-Preserved, TOPCAT and PARAGON-HF, each trial identified a potential benefit in the primary outcome: LVEF 40–49% (0.76 [0.61–0.96]); LVEF < 50% (0.72 [0.50–1.05]); and LVEF < 57% (0.78 [0.64–0.95]), respectively [39,40,41].

LVEF and the relationship between CV outcomes is a well-known and prognostic indicator [42,43,44]. Utilizing patients from the CHARM program, which included 7599 patients with heart failure and a mean LVEF of 38.8%, investigators identified a reduction in EF below 45% was independently associated with fatal and non-fatal CV outcomes. The same relationship was not identified as EF increased >45% and the risk was lower than in HFrEF. This is consistent with other studies identifying lower mortality rates with higher LVEFs. Thus, the availability of an LVEF is crucial in identifying high-risk HF patients and preventing adverse outcomes through the initiation of guideline-directed medical therapy (GDMT).

NT-proBNP (amino-terminal pro-B-type natriuretic peptide) and B-type natriuretic peptide (BNP) are useful biomarkers supported by guidelines to aid in the diagnosis of HF and risk stratification in chronic HF [45]. Higher levels of NT-proBNP and BNP have been associated with adverse outcomes in HF [46]. These cardiac biomarkers have been hypothesized to guide titration of HF therapies, which led to the GUIDE-IT trial [47]. The results of the study do not support titrating GDMT using NT-proBNP to prevent HHF or CV death in comparison to routine HF care.

Despite the evidence from randomized controlled trials establishing cardiovascular benefit from various medical therapies in HFrEF, these agents are often under-utilized or under-dosed [48,49]. There has been a shift from the historical paradigm of sequential therapy to simultaneously initiating quadruple therapy in HF. Strategies to improve therapy utilization have included the development of rapid therapy initiation algorithms, HF risk models, specialized uptitration clinics and tailored electronic health records alerts [50,51]. The EF classification system supports prompt identification of patients who will benefit greatly from GDMT.

### 4.2. Ejection Fraction as a Continuous Variable

We have identified numerous medication classes that have been found to improve mortality and CV benefit in HFrEF; however, there are limited pharmacological therapies targeted for HFpEF. One plausible answer is related to the heterogeneity of the HFpEF population. This was demonstrated with the differences in the baseline characteristics and HF etiology in I-Preserve and CHARM-Preserved. Lam et al. described six potential mechanisms to serve as potential therapies in HFpEF [52]. International societal guidelines even differ in their definitions of HFpEF, which often include symptoms, EF cutoffs and varying criteria such as elevated natriuretic peptides and echocardiogram evidence. The landmark trials TOPCAT, EMPEROR-Preserved and PARAGON-HF all varied in terms of EF threshold, cardiac enzyme elevations and presence of structural heart disease. Further characterization of HFpEF in order to better understand its pathophysiology and contributing comorbidities is crucial. While pre-specified analyses included in the trials are often completed, their results generate hypotheses that require further investigation. This is consistent with the regional differences in clinical outcomes as identified in the TOPCAT trial given the potential significant benefit of spironolactone in the Americas [53]. Further characterization of HFpEF in order to better understand its pathophysiology and contributing comorbidities is warranted before additional trials for therapies are conducted.

One class of medications, SGLT2i, are considered first-line agents in HF regardless of diabetic status. The DELIVER and DAPA-HF trials demonstrated reduction in cardiovascular death, hospitalization for heart failure, and major adverse cardiovascular events across all left ventricular ejection fractions. EMPEROR-Preserved is the first randomized control trial to reach the primary endpoint of HFpEF. The influence of EF in the pre-specified subgroups 41–49%, 50–59% and ≥60% did not change the primary endpoint. The observed clinical benefit with empagliflozin and dapagliflozin in both HFrEF and HFpEF was found to start within 12 to 28 days [54]. In light of the ejection fraction classification via echocardiogram, newly diagnosed or suspected HF patients could qualify for early initiation of SGLT2i to maximize their early benefit. This early benefit has also been demonstrated in other trials such as COPERNICUS, SOLVD, PARADIGM-HF and EMPHASIS-HF within the first 30 days of randomization [55].

For patients with favorable recovery in cardiac function who are asymptomatic, the benefit of HF therapies is unknown. The TRED-HF trial randomized patients to either continue or withdraw their HF treatment [56]. The primary endpoint (relapse of dilated cardiomyopathy, reduction in left ventricular ejection fraction, increase in left ventricular end-diastolic volume, increase in NT-pro-BNP, or clinical evidence of heart failure) was reached in 44% of patients that discontinued treatment, while none in the continuation of treatment arm reached the primary endpoint (Kaplan-Meier estimate of event rate 45.7% (95% CI 28.5–67.2); *p* = 0.0001). Similarly, Moon et al. followed over 40 patients with dilated cardiomyopathy and a recovered EF [57]. Of those, seven discontinued therapy and five had worsened LVEF. The withdrawal of these agents may have detrimental effects on cardiac function and demonstrate the potential ongoing benefit of these therapies despite the EF classification.

### 4.3. Prompt Initiation of Quadruple Heart Failure Therapies

With the well-studied time-to-benefit profiles of quadruple heart failure therapies (beta blocker, ARNI, SGLT2i, MRA), benefits can be quickly seen from these disease-modifying therapies in HFrEF patients [58]. Broadly speaking, the benefits of decreased mortality and hospitalization can be quickly observed in a few weeks after initiation of therapy. As such, prompt initiation of HF quadruple therapy is lifesaving. Despite strategies to expedite HF therapy initiation such as GDMT, clinical inertia remains [59]. This inertia is attributed in part to the perceived increase in adverse effects from medication. However, disease state worsening, adverse effects and death are more likely to occur from delaying the initiation of therapy. Greene et al. propose simultaneous or rapid-sequence initiation of quadruple therapy on day 1 in both hospitalized patients and outpatients as life-saving therapies [58]. In addition, the STRONG-HF trial demonstrated patient concordance that intensive treatment and rapid up-titration of GDMT improved quality of life and reduced mortality, readmission and symptoms [60].

## 5. Conclusions

Ejection fraction remains a useful clinical tool to classify patients with HF and predict mortality [42]. As definitions continue to evolve away from “diastolic” and “systolic” HF, we suspect that HF is on a continuous spectrum given the heterogeneity of the condition. This has significantly contributed to the difficulty of designing pragmatic, randomized controlled trials to identify pharmacological agents across the EF spectrum. However, SGLT2i have demonstrated a significant clinical benefit in HFpEF and HFmEF and we suspect these agents will continue to be promising therapeutic options to all HF patients. In conclusion, therapeutic strategies, regardless of EF, should be individualized to each patient.

## Figures and Tables

**Table 1:** Angiotensin-converting enzyme inhibitor heart failure trials.

Trial (Medication)	EF Inclusion	Major Outcome RR (95% CI) *p*-Value	Key Summary
CONSENSUS [5] (enalapril 2.5–40 mg daily)	≤40%	6-month death 0.60 *p* = 0.002	Enalapril reduced death in HFrEF patients.
SOLVD [6] (enalapril 2.5–20 mg daily)	≤35%	Death 0.84 (0.74–0.95) *p* = 0.0036	Enalapril reduced death and hospitalization in HFrEF patients.
SOLVD [7] (enalapril 2.5–20 mg daily)	≤40%	HF incidence 0.71 (0.64–0.79) *p* < 0.001	Enalapril reduced incidence of HF and HHF in asymptomatic reduced LVEF patients.
PEP-CHEF [8] (perindopril 2–4 mg daily)	>40%	Death and HHF 0.92 (0.70–1.21) *p* = 0.55	Perindopril did not reduce death or HHF in elderly patients.

HF: heart failure; HHF: hospitalization for heart failure; HFrEF: heart failure reduced ejection fraction; LVEF: left ventricular ejection fraction.

**Table 2:** Angiotensin receptor blocker heart failure trials.

Trial (Medication)	EF Inclusion	Major Outcome RR/HR (95% CI) *p*-Value	Key Summary
ELITE [9] (losartan 12.5 to 50 mg daily or captopril 6.25 to 50 TID)	≤40%	Death 0.54 (0.05–0.69) *p* = 0.0035	In elderly HFrEF patients, losartan reduced more death than captopril.
RESOLVD [9] (candesartan 4 to 16 mg or enalapril 20 mg)	≤40%	Combination therapy reduced aldosterone (*p* < 0.05) and brain natriuretic peptide (5.8 ± 2.7 pmol/L; *p* < 0.01)	Combination of regimen was more effective in preventing left ventricular remodeling.
ELITE II [10] (losartan 12.5 to 50 mg or captopril 12.5 to 50 mg TID)	≤40%	Difference in death 1.13 (0.95–1.35) *p* = 0.16	Losartan was not superior to captopril in improving survival but was better tolerated.
Val-HeFT [11] (valsartan 160 mg daily)	<40%	Composite of morbidity, death 0.87 (0.77–0.97) *p* = 0.009	Valsartan reduced morbidity and death.
CHARM-Overall [12] (candesartan 4 to 32 mg daily)	<40%	CV death 0.88 (0.79–0.97) *p* = 0.012	Candesartan reduced CV death and HHF in HFrEF.
CHARM-Added [12] (candesartan 4 to 32 mg daily)	<40%	CV death or HHF 0.85 (0.75–0.96) *p* = 0.011	Adding candesartan to ACEi HF therapy reduced CV death or HHF.
CHARM-Alternative [13] (candesartan 4 to 32 mg daily)	<40%	CV death or HHF 0.77 (0.67–0.89) *p* = 0.0004	In HFrEF patients not taking ACEi, candesartan reduced CV death and HHF.
CHARM-Preserved [14] (candesartan 32 mg)	>40%	HHF 0.84 (0.70–1.00) *p* = 0.047	Candesartan reduced HHF, but not CV death.
I-Preserve [15] (irbesartan 300 mg daily)	>45%	Death or CV hospitalization 0.95 (0.86–1.05) *p* = 0.35	Irbesartan did not reduce death or CV hospitalization in HFpEF patients.

ACEi: angiotensin-converting enzyme inhibitors; CV: cardiovascular; HF: heart failure; HHF: hospitalization for heart failure; HFpEF: heart failure preserved ejection fraction; HFrEF: heart failure reduced ejection fraction; TID: three times a day.

**Table 3:** Sacubitril–valsartan heart failure trials.

Trial (Medication)	EF Inclusion	Major Outcome HR (95% CI) *p*-Value	Key Summary
PARADIGM [16] (sacubitril-valsartan 97/103 mg BID)	≤40% (until 2010 then reduced to ≤35%)	Composite CV death of HHF 0.80 (0.73–0.87) *p* < 0.001	Sacubitril-valsartan superior to enalapril in reducing death and HHF
PARAGON-HF [17] (sacubitril-valsartan 97/103 mg BID)	≥45%	Composite of CV death and HHF 0.87 (0.75–1.01) *p* = 0.059	Sacubitril-valsartan did not significantly reduce HHF or CV death in patients with EF of 45% or greater
PIONEER-HF [18] (sacubitril-valsartan 24/26 mg BID—97/103 mg BID)	≤40%	NT-pro-BNP values and time-averaged change from baseline 0.71 (0.63–0.81) *p* < 0.001	Sacubitril-valsartan significantly reduced NT-proBNP in HFrEF patients admitted with ADHF
PARADISE-MI [19] (sacubitril–valsartan 97/103 mg BID)	≤40%	Time to first composite endpoint including CV death, HHF or outpatients 0.90 (0.78–1.04) *p* = 0.17	Sacubitril-valsartan not associated with superior CV endpoints in the AMI population
PROVE-HF [20] (sacubitril–valsartan 24/26 mg BID—97/103 mg BID)	≤40%	NT-pro-BNP values and time-averaged change from baseline 0.85 (0.77–0.94) *p* < 0.001	Reductions in NT-proBNP may improve cardiac function and volume with sacubitril-valsartan

ADHF: acute decompensated heart failure; AMI: acute myocardial infarction; CV: cardiovascular; EF: ejection fraction; HFrEF: heart failure reduced ejection fraction; HHF: heart failure for hospitalization; NT-proBNP: N-terminal pro-brain natriuretic peptide.

**Table 4:** Beta blocker heart failure trials.

Trial (Medication)	EF Inclusion	Major Outcome HR (95% CI) *p*-Value	Key Summary
MERIT-HF [21] (metoprolol 12.5 or 25 mg)	≤40%	All-cause mortality: relative risk 0.66 [95% CI 0.53–0.81]; *p* = 0.00009	Metoprolol in addition to standard therapy improved survival
CIBIS-II [22] (bisoprolol 1.25–10 mg daily)	≤35%	All-cause mortality: 0.66 (95% CI 0.54–0.81) *p* < 0.0001	Adding BB to standard therapy (diuretics, ACEi) had benefits for survival
COPERNICUS [23] (carvedilol 3.125 mg BID—25 mg BID)	≤25%	Mortality (RR 0.65; 95% CI 0.52–0.81; *p* = 0.00013; NNT = 15)	Carvedilol reduced risk of death or HF hospitalization
US Carvedilol [24] (carvedilol 6.25–50 mg BID)	≤35%	Mortality (7.8% in placebo, 3.2% in carvedilol group)	Carvedilol reduced death and hospitalization

ACEi: angiotensin-converting enzyme inhibitors; BB: beta blocker; BID: twice daily; HF: heart failure.

**Table 5:** Mineralocorticoid receptor antagonist heart failure trials.

Trial (Medication)	EF Inclusion	Major Outcome HR (95% CI) *p*-Value	Key Summary
TOPCAT [25] (spironolactone 15–45 mg daily)	≥45%	CV mortality, aborted cardiac arrest or HHF 0.89 (0.77–1.04) *p* = 0.14	Spironolactone did not reduce CV composite in HFpEF
RALES [26] (spironolactone 25 mg daily)	≤35%	Death 0.70 (0.60–0.82) *p* < 0.001	Spironolactone reduced morbidity, death in severe HF
EPHESUS [27] (eplerenone 25–50 mg daily)	≤40%	Death from any cause; CV death or first hospitalization for a CV event 0.85 (0.75–0.960 *p* = 0.008 and 0.87 (0.79–0.95) *p* = 0.002	Eplerenone reduced mortality among acute MI with HF symptoms
EMPHASIS-HF [27] (eplerenone 25–50 mg daily)	≤30%	CV death or HF hospitalization 0.63 (0.54–0.74) *p* < 0.001	Eplerenone reduced risk of death and hospitalization in HFrEF

CV: cardiovascular; HF: heart failure; HFpEF: heart failure with preserved ejection fraction.

**Table 6:** Ivabradine heart failure trials.

Trial (Medication)	EF Inclusion	Major Outcome HR (95% CI) *p*-Value	Key Summary
SHIFT [29] (ivabradine 5–7.5 mg BID)	≤35%	HHF or CV death 0.82 (0.75–0.90) *p* < 0.0001	Ivabradine decreased the risk of CV death or HHF with a resting HR > 70 bpm
EDIFY [30] (ivabradine 5–7.5 mg BID)	≥45%	Echo Doppler E/e’ ratio, 6MWT and plasma NT-proBNP concentration	Ivabradine did not improve co-primary endpoints in HFpEF with a resting HR > 70 bpm

BID: twice daily; Bpm: beats per minute; HHF: heart failure hospitalization; HR: heart rate; 6MWT: 6 min walking test; E/e’: mitral inflow velocity over early diastolic mitral annular velocity.

**Table 7:** Sodium Glucose Cotransporter 2 Inhibitor heart failure trials.

Trial (Medication)	EF Inclusion	Major Outcome HR (95% CI) *p*-Value	Key Summary
DAPA-HF [31] (dapagliflozin 10 mg daily)	≤40%	Composite of worsening HF or CV death 0.74 (0.65–0.85) *p* < 0.001	Dapagliflozin lowered the risk of worsening HF or CV death in HFrEF patients, regardless of diabetic status
EMPEROR-Reduced [32] (empagliflozin 10 mg daily)	≤40%	Composite CV death or HHF 0.75 (0.65–0.86) *p* < 0.001	Empagliflozin reduced CV death and HHF in HFrEF regardless of absence or presence of diabetes
EMPEROR-Preserved [33] (empagliflozin 10 mg daily)	≥40%	Composite of CV death or HHF 0.79 (0.69–0.90) *p* < 0.001	Empagliflozin reduced CV death or HHF in HFpEF patients
SOLOIST-WHF [34] (sotagliflozin 200 or 400 mg daily)	Presence of signs and symptoms of HF	CV death and HHF 0.67 (0.52–0.85) *p* < 0.001	This was the first large trial of SGLT1/SGLT2 inhibitor in hospitalized patients
DELIVER [38] (dapagliflozin 10 mg daily)	≥40% or previously <40% but recovered	Composite of worsening HF or CV death 0.82 (0.73–0.92) *p* < 0.001	Dapagliflozin reduced the combined risk of worsening heart failure or cardiovascular death among patients with heart failure and a mildly reduced or preserved ejection fraction

CV: cardiovascular; HF: heart failure; HHF: hospitalization for heart failure; HFpEF: heart failure preserved ejection fraction; HFrEF: heart failure reduced ejection fraction; SGLT1: Sodium Glucose Cotransporter 1; SGLT2: Sodium Glucose Cotransporter 2.

## Data Availability

Not applicable.
